# Stonikacidin A, an Antimicrobial 4-Bromopyrrole Alkaloid Containing L-Idonic Acid Core from the Northwestern Pacific Marine Sponge *Lissodendoryx papillosa*

**DOI:** 10.3390/md22090396

**Published:** 2024-08-30

**Authors:** Kseniya M. Tabakmakher, Tatyana N. Makarieva, Yuri E. Sabutski, Maxim S. Kokoulin, Alexander S. Menshov, Roman S. Popov, Alla G. Guzii, Larisa K. Shubina, Ekaterina A. Chingizova, Artur R. Chingizov, Ekaterina A. Yurchenko, Sergey N. Fedorov, Boris B. Grebnev, Gunhild von Amsberg, Sergey A. Dyshlovoy, Natalia V. Ivanchina, Pavel S. Dmitrenok

**Affiliations:** 1 G.B. Elyakov Pacific Institute of Bioorganic Chemistry, Far Eastern Branch of the Russian Academy of Sciences, Pr. 100-let Vladivostoku 159, 690022 Vladivostok, Russia; tabakmakher_km@piboc.dvo.ru (K.M.T.); alixar2006@gmail.com (Y.E.S.); maxchem@mail.ru (M.S.K.); menshov90@piboc.dvo.ru (A.S.M.); popov_rs@piboc.dvo.ru (R.S.P.); gagry@rambler.ru (A.G.G.); shubina@piboc.dvo.ru (L.K.S.); chingizova_ea@piboc.dvo.ru (E.A.C.); chingizov84@gmail.com (A.R.C.); eyurch@piboc.dvo.ru (E.A.Y.); fedorov@pidoc.dvo.ru (S.N.F.); grebnev_bor@mail.ru (B.B.G.); ivanchina@piboc.dvo.ru (N.V.I.); paveldmt@piboc.dvo.ru (P.S.D.); 2 Department of Oncology, Hematology and Bone Marrow Transplantation with Section Pneumology, Hubertus Wald Tumorzentrum–University Cancer Center Hamburg (UCCH), University Medical Center Hamburg-Eppendorf, 20251 Hamburg, Germany; g.von-amsberg@uke.de (G.v.A.); dyshlovoy@gmail.com (S.A.D.); 3 Martini-Klinik, Prostate Cancer Center, University Hospital Hamburg-Eppendorf, 20251 Hamburg, Germany

**Keywords:** *Lissodendoryx papillosa*, bromopyrrole alkaloid, idonic acid, antimicrobial activity

## Abstract

Stonikacidin A (**1**), the first representative of a new class of 4-bromopyrrole alkaloids containing an aldonic acid core, was isolated from the marine sponge *Lissodendoryx papillosa*. The compound is named in honor of Prof. Valentin A. Stonik, who is one of the outstanding investigators in the field of marine natural chemistry. The structure of **1** was determined using NMR, MS analysis, and chemical correlations. The L-idonic acid core was established by the comparison of GC, NMR, MS, and optical rotation data of methyl-pentaacetyl-aldonates obtained from the hydrolysis products of **1** and standard hexoses. The L-form of the idonic acid residue in **1** was confirmed by GC analysis of pentaacetate of (*S*)-2-butyl ester of the hydrolysis product from **1** and compared with corresponding derivatives of L- and D-idonic acids. The biosynthetic pathway for stonikacidin A (**1**) was proposed. The alkaloid **1** inhibited the growth of *Staphylococcus aureus* and *Escherichia coli* test strains, as well as affected the formation of *S. aureus* and *E. coli* biofilms. Compound **1** inhibited the activity of sortase A. Molecular docking data showed that stonikacidin A (**1**) can bind with sortase A due to the interactions between its bromine atoms and some amino acid residues of the enzyme.

## 1. Introduction

Marine sponges are a rich source of biologically active secondary metabolites of various structural classes [1]. Among them, bromopyrrole alkaloids occupy a prominent position. They represent an attractive example of the wide diversity of metabolites produced by marine sponges, which exhibit various biological activities, including antiangiogenic, anti-Alzheimer, antibacterial, antibiofilm, anticancer, antifeedant, antifouling, antifungal, antihistaminic, anti-inflammatory, antimicrobial, antimuscarinic, antiparasitic, antiviral, enzyme inhibitory, immunosuppressive, neuroprotective, somatostatin inhibitory and other [2,3,4,5,6,7,8,9,10,11,12,13]. These compounds are found in a number of marine sponges of the class Demospongiae, as well as in some representatives of Bryozoans [2]. Bromopyrrole alkaloids were previously considered as a taxon-specific marker for marine sponges of the order Agelasida; however, later, they were also found in representatives of other orders of marine sponges, such as Axinellida, Bubarida, Halichondrida, Haplosclerida, Scopalinida, and Verongida [14,15,16,17,18,19,20]. The structural diversity of compounds in this class is associated with the number of pyrrole rings in the molecule, the number and position of bromine atoms, as well as the structure of the core, usually represented by a nitrogen-containing moiety, to which the bromopyrrole residues are attached. A characteristic feature of these metabolites is that the bromopyrrole residues are connected to the core through amide bonds, and only some of them have an ester bond instead of an amide one [21,22,23,24].

In continuation of our search for bioactive natural products from the Northwestern Pacific marine sponges [25] we found that ethanolic extract of the marine sponge *L. papillosa*, collected near Urup Island (Kurile Islands, 45°35′5″ N; 149°47′7″ E, a depth of 145 m), strongly inhibited the growth of *S. aureus* ATCC 21,027 and *E. coli* VKPM (B-7935). Bioassay-guided fractionation led to the isolation of a new natural bromopyrrole alkaloid named stonikacidin A (**1**). The compound is named in honor of Prof. Valentin A. Stonik (born in 1942), who is one of the outstanding investigators in the field of marine natural chemistry. Together with his collaborators, he described several hundred new natural compounds, particularly from echinoderms, sponges, and other marine organisms.

Marine sponges of the genus *Lissodendoryx* have been poorly studied chemically, but there is information that structurally diverse compounds, both nitrogen- and non-nitrogen-containing, were found in the studied species of this genus [1] (Supplementary Materials, list of previously isolated metabolites from the sponges of the genus *Lissodendoryx*). These metabolites showed a wide range of biological activities, including a potent capability to reduce the production of reactive oxygen species in Neuro 2a neuroblastoma cells and modestly increase the survival of these cells upon treatment with 6-hydroxydopamine (an in vitro anti-Parkinson’s biotest) for manzamine-related alkaloids from the Far Eastern sponge *Lissodendoryx florida* [26]. Manadosterols A and B, sulfonated sterol dimers isolated from the North Sulawesi marine sponge *Lissodendryx fibrosa,* inhibited the Ubc13−Uev1A interaction [27]. Cembranes from the Antarctic *Lissodendoryx flabellate* were cytotoxic to Nl8-T62 mouse neuroblastoma cells at low concentrations (0.16 µM) and significantly reduced cell proliferation of human tumor cells DU-145 and MCF-7 [28].

This work is the first report of a chemical investigation of the marine sponge *L. papillosa*. Here, we report the structure and bioactivity of stonikacidin A (**1**), an unprecedented bromopyrrole-containing compound with an acyclic carbohydrate acid core.

## 2. Results and Discussion

### 2.1. Isolation and Structure Elucidation

**Figure 1:**
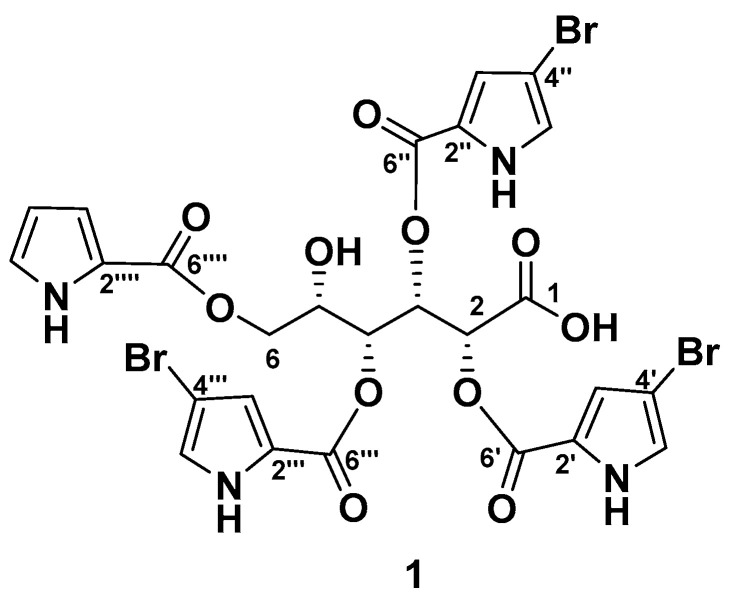
The chemical structure of stonikacidin A (**1**).

The EtOH extract of the frozen sponge *L. papillosa* was partitioned between H_2_O and *n*-BuOH, and the *n*-BuOH-soluble material was separated by reversed-phase flash column followed by reversed-phase HPLC chromatography to give stonikacidin A (**1**) (Figure 1).

The molecular formula of **1** was determined to be C_26_H_21_Br_3_N_4_O_11_ based on the deprotonated molecule ion peak at *m/z* 800.8683 [M–H]^−^ (calcd for [C_26_H_20_^79^Br_3_N_4_O_11_]^−^ *m/z* 800.8684), and an adduct ion at *m/z* 846.8483 [M + 2Na–H]^+^ (calcd for [C_26_H_20_^79^Br_3_N_4_O_11_Na_2_]^+^ *m/z* 846.8469) in the HRESIMS spectra. The characteristic isotopic distribution of molecular ions in **1** confirmed the presence of three bromine atoms (Figures S1–S7, Tables S1 and S2).

The NMR spectra of **1** (Table 1, Figures S8–S12) revealed resonances of twelve nonprotonated carbons (*δ*_C_ 98.0, 98.1, 98.3, 122.9, 123.3, 123.5, 123.6, 160.1, 160.6, 160.7, 162.1, 172.8), nine aromatic methines (*δ*_H_ 6.12, 6.67, 6.76, 6.80, 6.88, 6.90, 6.91, 6.98, 7.01; *δ*_C_ 110.8, 117.2, 118.8, 118.9, 124.7, 125.03, 124.9, 119.0, 125.05), four mutually coupled oxygenated methines (*δ*_H_ 4.27, 5.61, 5.65, 6.27; *δ*_C_ 68.4, 73.0, 73.6, 72.8) and a methylene group (*δ*_H_ 4.10, 4.28; *δ*_C_ 64.6).

**Figure 2:**
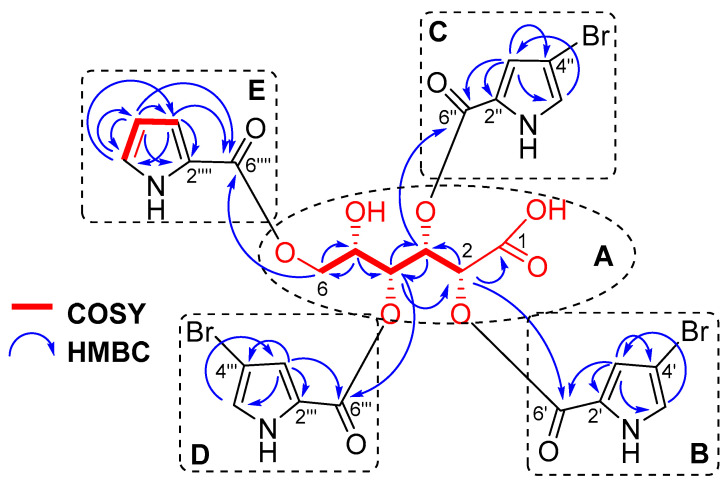
COSY and HMBC correlations of stonikacidin A (**1**).

The chemical shift of the carbonyl at *δ*_C_ 172.8, together with an IR absorption band at 1703 cm^−1^ (Figure S13), indicated the presence of a carboxyl group in **1** consistent with the molecular formula. The HMBC cross-peak H-2 (*δ*_H_ 5.65)/C-1 (*δ*_C_ 172.8) and the COSY correlations from H-2 to H_2_-6 confirmed an aldonic acid core in **1** (Figure 2**A**, Figure S10 and S12).

The 4-bromopyrrole-2-carboxylic acid residues were evident from the NMR data of **1** (Table 1, Figure 2**B**–**D** and Figure S8–S12), which are comparable with those reported in the literature for other bromopyrrole alkaloids [23,29,30]. The MS/MS spectrum of the ion [M + 2Na–H]^+^ in **1** produced fragment ion peaks at *m/z* 657.9058 [M + 2Na–H–C_5_H_4_^79^BrNO_2_]^+^, 468.9630 [M + 2Na–H–2×C_5_H_4_^79^BrNO_2_]^+^, and 280.0195 [M + 2Na–H–3×C_5_H_4_^79^BrNO_2_]^+^ (Figures S6 and S7). These data confirmed the presence of three 4-bromopyrrole-2-carboxylic acid moieties. The pyrrole-2-carboxylic acid unit was established based on the COSY correlations of aromatic protons from H-3⁗ to H-5⁗ and HMBC cross-peaks H-3⁗/C2⁗, C4⁗, C6⁗; H-4⁗/C2⁗, C3⁗, C5⁗, C6⁗; and H-5⁗/C3⁗, C4⁗ (Figure 2**E**, Figure S10). The HMBC correlations H-2 (*δ*_H_ 5.65)/C-6′ (*δ*_C_ 160.7), H-3 (*δ*_H_ 6.27)/C-6″ (*δ*_C_ 160.1), H-4 (*δ*_H_ 5.61)/C-6‴ (*δ*_C_ 160.7), H-6a (*δ*_H_ 4.10), and H-6b (*δ*_H_ 4.28)/C-6⁗ (*δ*_C_ 162.1) supported the placement of 4-bromopyrrole-2-carboxylic acid residues at C-2, C-3, and C-4, and a pyrrole-2-carboxylic moiety at C-6 (Figure S12). The shielded chemical shift of H-5 (*δ*_H_ 4.27), as well as HMBC data, showed the location of the hydroxyl group at C-5.

The exact determination of the relative stereochemistry of the aldonic acid core in **1** was not possible by NMR analysis due to its acyclic structure. The transformation of **1** to iditol was not relevant due to the small amount of isolated substance.

To determine the configurations of the asymmetric center of the acyclic aldonic acid core, stonikacidin A (**1**) was hydrolyzed with MeONa, followed by acetylation (Scheme 1). These reactions yielded **2**, the structure of which was confirmed by HRESIMS, NMR data, and GC analysis in comparison with corresponding data for similar derivatives obtained from standard monosaccharides (**3a**–**3f**) (Experimental Section, Schemes S1–S3, Figures S16–S30, Table S3). GC analysis of **2** by co-injection with standard monosaccharide derivatives revealed the presence of an idonic acid core in **1** (Figures S31–S37).

The relative *ido*-configuration of the aldonic acid core in **1** was established based on the agreement of the GC and NMR data of derivative **2** with the corresponding data of the standard methyl-pentaacetyl-D-idonate (**6a**) (Scheme S3, Figures S18, S19, S31, S32, Table S3). However, compound **2** and methyl-pentaacetyl-D-idonate (**6a**) showed opposite signs of optical rotation data (${\left[\alpha \right]}_{D}^{20}$−17 for **2** and ${\left[\alpha \right]}_{D}^{20}$+21 for **6a**, respectively). Consequently, compound **2** was proposed to be methyl-pentaacetyl-L-idonate.

The oxidation, acetylation, and methylation of L-idose synthesized from the 1,2-isopropylidene derivative of D-glucuronic acid γ-lactone (Scheme S2) yielded the methyl-pentaacetyl-L-idonate (**6f**) (Scheme S3). Comparison of the optical rotation data of **2** with that of **6f** (${\left[\alpha \right]}_{D}^{20}$−19) suggested the same L-absolute configuration in the idonic acid core of **1**.

The L-configuration of the idonic acid core in stonikacidin A (**1**) was confirmed by preparation of the (*S*)-2-butyl ester of **2,** followed by acetylation and GC analysis in comparison with the corresponding standard L- and D-idonate derivatives (Experimental Section, Figure S38).

Thus, stonikacidin A (**1**) contains an L-idonic acid core substituted by three 4-bromopyrrole-2-carboxylic acids and one pyrrole-2-carboxylic acid residue.

### 2.2. Proposed Biosynthetic Pathways for the Formation of Stonikacidin A (***1***)

The structure of stonikacidin A (**1**) suggests its biogenesis from a D-glucose (**I**) through interaction by an invertase (Scheme 2). Earlier, glucose invertase (GI) from *Streptomyces griseofuscus* was found to exhibit activity toward D-glucose [31,32,33]. Additional biosynthetic conversions might include the oxidation of L-idose (**II**) to obtain idonic acid (**III**) and, after selective acylation by pyrrole-2-carboxylic acid, to form a putative intermediate (**IV**). Selective bromination by bromoperoxydase [34] could complete the process of biosynthesis of stonikacidin A (**1**).

### 2.3. Study of the Biological Activity of Stonikacidin A (**1**)

#### 2.3.1. Antimicrobial Assay

**Figure 3:**
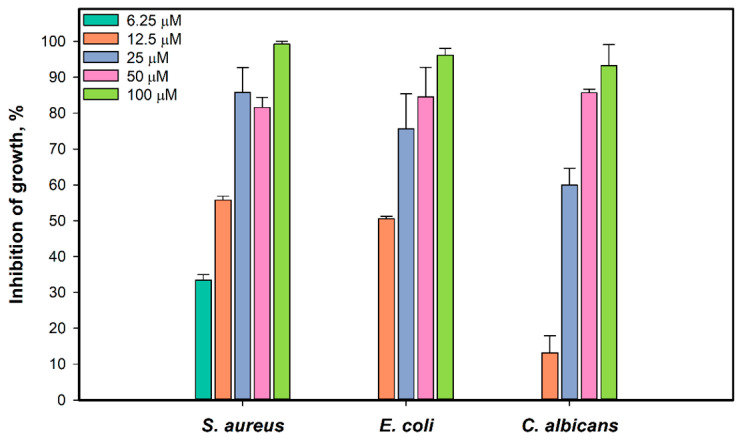
Effect of **1** on the growth of test strains *S. aureus*, *E. coli*, and *C. albicans*. All experiments were carried out in triplicate, and data are presented as mean ± standard error of the mean (SEM).

We investigated the antimicrobial activity of **1** against the growth and biofilm formation of *S. aureus* ATCC 21027, *E. coli* VKPM (B-7935), and *Candida albicans* KMM 455 test strains. The effect of **1** on the growth of *S. aureus*, *E. coli,* and *C. albicans* after incubation for 18 h is presented in Figure 3. All test strains were completely inhibited by **1** at a concentration of 100 µM. Compound **1,** at a concentration of 6.25 µM, inhibited the growth of *S. aureus* by 33.5%, and the half-maximal concentration (IC_50_) was calculated as 10.9 ± 1.2 µM. The growth of *E. coli* was inhibited by 50.6% at a concentration of **1** of 12.5 µM. The IC_50_ of growth inhibition of *C. albicans* was calculated as 22.3 µM.

**Figure 4:**
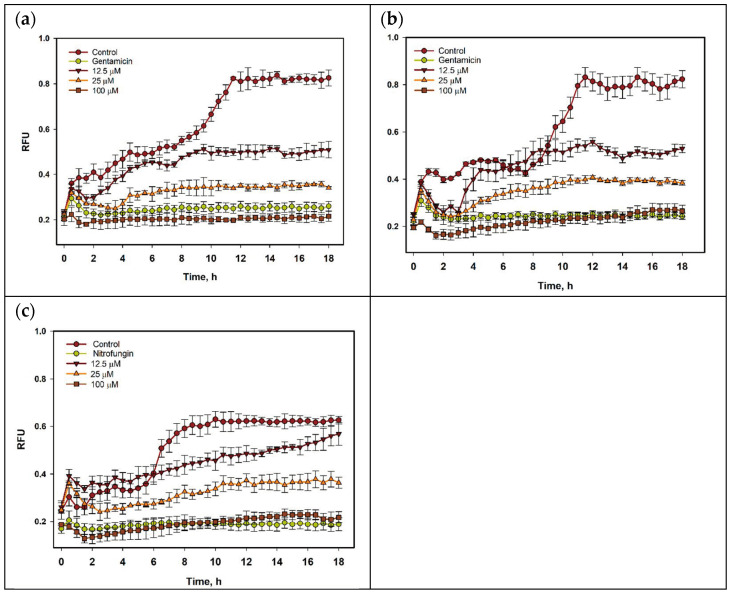
The growth of *S. aureus* (**a**), *E. coli* (**b**), and *C. albicans* (**c**) treated with compound **1** within 18 h. All experiments were carried out in triplicate, and data are presented as mean ± standard error of the mean (SEM).

The time-dependent effect of compound **1** on the growth of *S. aureus*, *E. coli*, and *C. albicans* within 18 h is presented in Figure 4. The growth-inhibiting influence of **1** was detected after 1 h of co-incubation and was stable during all periods of observation. The growth of bacterial suspensions after 8 h was enhanced, but only **1** was bacteriostatic at all investigated concentrations. However, the inhibiting effect of **1** on the growth of *C. albicans* was less.

**Figure 5:**
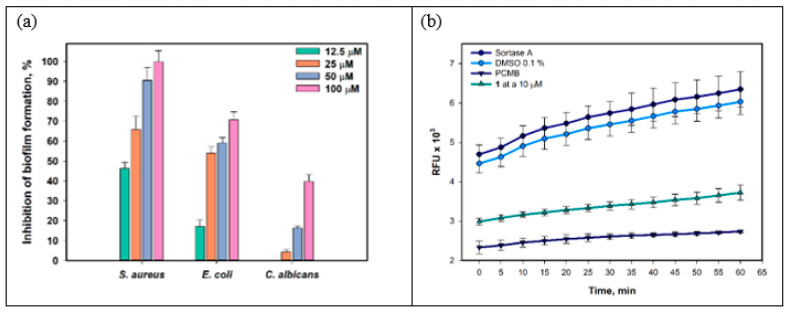
Effect of **1** on biofilm formation of *S. aureus*, *E. coli,* and *C. albicans* (**a**). Effect of **1** at a concentration of 10 µM on the activity of the enzyme sortase A within 60 min (**b**). All experiments were carried out in triplicate, and data are presented as mean ± standard error of the mean (SEM).

The effect of compound **1** on the formation of biofilms of *S. aureus*, *E. coli*, and *C. albicans* is presented in Figure 5a. Compound **1** inhibited the formation of *S. aureus* biofilms by 46.3% at a concentration of 12.5 µM, and the IC_50_ was calculated as 14.9 µM. *E. coli* biofilm formation was inhibited by 17.1% under compound **1** at a concentration of 12.5 µM, and the IC_50_ was calculated as 23.6 µM. Biofilm formation of *C. albicans* was inhibited by 16.4% and 39.7% only at the compound concentrations of 50 µM and 100 µM, respectively.

Thus, a significant effect of **1** on the biofilm formation of *S. aureus* was found. Biofilm formation is a multifactorial process involving adhesion to the surface and the development of bacterial microcolonies into mature biofilm [35]. Various elements of quorum sensing systems are involved in biofilm formation, and targeting them can lead to the prevention or destruction of biofilms. We studied the effect of **1** on the activity of the enzyme sortase A, which is one of the key factors in the formation of *S. aureus* biofilm [36]. The data are presented in Figure 5b. Compound **1** at a concentration of 10 µM was measured to inhibit sortase A activity by 38.8–41.4%. It should be noted that increasing the concentration of compound **1** to 50 µM did not increase the inhibition of sortase A activity.

**Figure 6:**
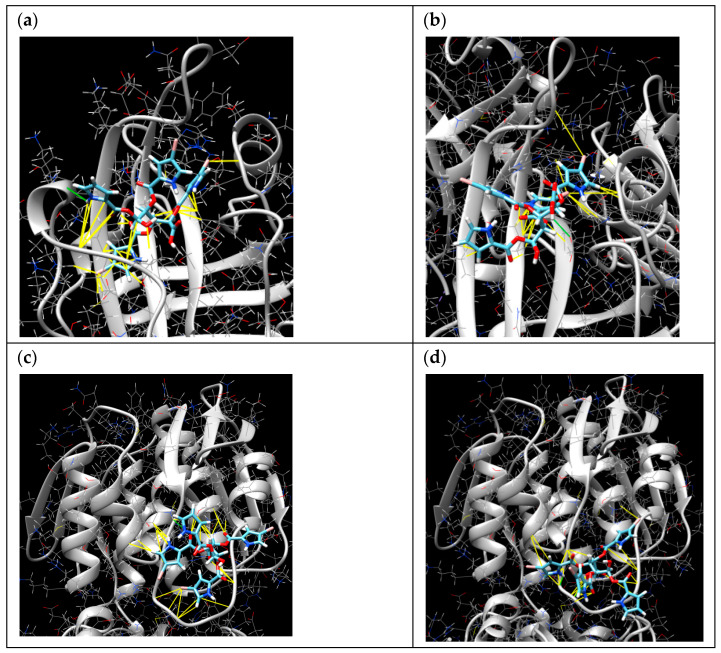
The molecular docking calculated poses of **1** with sortase A (**a**,**b**) and MurA (**c**,**d**). The yellow lines indicate the hydrophobic interactions and the green lines indicate the hydrogen bindings.

The molecular docking online server SwissDock was used for the calculation of interactions of **1** with sortase A (PDB 1T2P) and UDP-N-acetylglucosamine enolpyruvyl transferase (MurA) (PDB ID 1UAE). The poses in active sites with minimal energies are presented in Table 2 and Figure 6. The pose of **1** with sortase A with ∆G of −8.939546 kcal/mol (Figure 6a) was calculated with hydrogen binding with Pro163 and hydrophobic interactions with Gly167, Ile199, Ala104, Ile182, Leu169, and Thr180 amino acid residues. Moreover, the complex has hydrophobic interactions between the bromine atom and Ala92 and other bromine atoms and Val201, Val166, and Val168. The second pose of compound **1** with sortase A with ∆G of −8.263234 kcal/mol (Figure 6b) was calculated with hydrogen binding with Glu105 and hydrophobic interactions with Cys184, Ala92, Ala104, Ile182, and Ile199. The complex has hydrophobic interactions between bromine atoms and Ala92 and Leu97 and other bromine atoms and Cys184, Gly192, and Trp194.

The pose of **1** with UDP-N-acetylglucosamine enolpyruvyl transferase (MurA) with ∆G of −7.1534557 kcal/mol (Figure 6c) was calculated with hydrogen binding with Lys88 and hydrophobic interactions between bromine atoms and both Cys115 and Thr166 as well as with Gly118, Lys88, Ala119, and Leu111. The second pose of **1** with MurA with ∆G of −8.188495 kcal/mol (Figure 6d) was calculated with hydrogen bindings with Cys115 and Gly114 as well as hydrophobic interactions with Leu111 (and bromine), Gly114, Lys88, Gly113, Ala119, and Gly118.

Earlier, a number of brominated compounds were isolated from different marine sponges and discovered as antimicrobial agents [37]. Moreover, some brominated compounds were reported as inhibitors of staphylococcal biofilm formation, and some of them inhibited the sortase A activity. For instance, the bisindole alkaloid 2,2-bis(6-bromo-3-indolyl)ethylamine isolated from the Californian tunicate, *Didemnum candidum,* and the New Caledonian sponge, *Orina* spp., inhibited the formation of *S. aureus* biofilms to a greater extent than *E. coli* and other strains [38]. The brominated pyrrole-imidazole alkaloids oroidin, sceptrin, and bromoageliferin from sponges of the family Agelasidae and their synthetic derivatives were discovered as antifouling and later as anti-biofilm agents [39]. Cadiolide E, a 4-(3-bromo-4-hydroxyphenyl)-2-furanone derivative, as well as isocadiolides A–D with tris-bromohydroxyphenyl moieties from a Korean dark red ascidian *Synoicum* sp. exhibited antibacterial effects and a significant inhibition on Sa-SrtA [40,41].

Molecular docking data showed that compound **1** can bind to sortase A through interactions between bromine atoms and certain amino acid residues. Particular attention should be paid to the calculated interaction of bromine atoms with Cys184. This amino acid residue, together with Arg197, forms the ligand binding site of sortase A [42], and its blocking leads to the inactivation of sortase A [43]. Despite the fact that various low molecular weight compounds (including bromine-containing ones) were evaluated as inhibitors of sortase A [44], there are no data on their interaction with the structure of sortase A.

The bromine atom in N-(2-bromo-4,4-dimethyl-3-oxocyclobut-1-en-1-yl)-N-methylbenzamide was found to be crucial for targeting the bacterial MurA [45]. The authors discovered the cysteine specificity of this brominated compound in a series of convincing experiments using glutathione and an oligopeptide, and MS/MS experiments proved covalent binding of the compound to Cys115 in MurA and the observed loss of the bromine atom suggests a net nucleophilic substitution as a covalent reaction. The MurA enzyme is essential for the synthesis of *E. coli* peptidoglycan and catalyzes the first step in bacterial cell wall biosynthesis. Cys115 forms the active site of this enzyme and is targeted by various antibiotics, including fosfomycin [46]. Molecular docking data showed that **1** can also form stable poses with MurA at the active site. Molecular docking data, together with analysis of the activity of cell-free sortase A and literature data, give us reason to assume that compound **1** is able to covalently bind the Cys184 residue in sortase A or Cys155 in MurA. However, these speculations, of course, need to be verified.

Thus, compound **1** was discovered as a promising antimicrobial agent that inhibited the growth and biofilm formation of both *S. aureus* and *E. coli* and targeted microbial cysteine enzymes.

#### 2.3.2. Cytotoxic Activity

We evaluated the cytotoxic activity of stonikacidin A (**1**) in a panel of twelve human cell lines, which included five non-cancerous cell lines MRC-9, HUVEC, HEK293T, PNT2, and RWPE-1, as well as seven prostate cancer cell lines PC3, PC3-DR, DU145, DU145-DR, 22Rv1, VCaP, and LNCaP [47]. Compound **1** did not show cytotoxicity in any of the cell lines at concentrations up to 50 μM. Analysis of the effect of **1** on p-gp activity was performed using a calcein-AM-based assay. P-gp-overexpressing PC3-DR cells with the established p-gp inhibitor tariquidar were used as a positive control [48]. We showed that compound **1** inhibits p-gp activity, as indicated by an increase in intracellular calcein retention (Figure S39).

## 3. Materials and Methods

### 3.1. General Procedures

All reagents were obtained from commercial suppliers and were used without additional purification. All solvents were distilled before use. Melting points were determined by using a Boetius apparatus (VEB Analytic, Dresden, Germany) and are uncorrected. Optical rotations were measured using a Perkin–Elmer 343 polarimeter. IR spectra were recorded using a Bruker Equinox 55 spectrophotometer. The ^1^H and ^13^C NMR spectra were recorded on a Bruker DRX 500 spectrometer at 500.13 and 125.76 MHz and a Bruker Avance III-700 spectrometer at 700.13 and 176.04 MHz, respectively. Chemical shifts were referenced to the corresponding residual solvent signal (δ_H_ 3.30/δ_C_ 49.60 for CD_3_OD and δ_H_ 7.26/δ_C_ 77.16 for CDCl_3_). Structural assignments were made with additional information from COSY, HSQC, and HMBC experiments. MS data were obtained using a Bruker maXis Impact II Q-TOF mass spectrometer (Bruker Daltonics, Bremen, Germany) by direct infusion of the sample solution in MeOH (C 0.05 mg/mL) in an ESI ionization source. GC analysis of methyl-pentaacetyl-aldonates was conducted on an Agilent 6580 Series (Agilent Technologies, Santa Clara, CA, USA) apparatus equipped with an HP-1 MS capillary column (30 m × 0.25 mm) with He as carrier gas (1.7 mL/min) using a temperature gradient of 100→250 °C at 5 °C/min. The temperatures of the injector and detector were 270 and 300 °C, respectively. GC analysis of pentaacetate of (S)-2-butyl ester derivatives was conducted on a Hewlett Packard 5890 chromatograph (Conquer Scientific, Poway, CA, USA) equipped with a Hewlett Packard 5973 mass spectrometer (Conquer Scientific, Poway, CA, USA) and an HP-5MS capillary column using the following temperature program: 150 °C for 3 min, then 150 °C → 290 °C at 3 °C/min, and 290 °C for 10 min. Low-pressure liquid column chromatography was performed using a YMC*Gel ODS-A column. HPLC was performed using an Agilent Series 1100 Instrument equipped with a differential refractometer RIDDE14901810 and a YMC-Pack ODS-A (5 μm, 250 × 10 mm) column and Supelco Discovery HS-F5-5 (5 μm, 250 × 10 mm) column and Supelco LCSI SEMI-PREP (5 μm, 250 × 10 mm) column.

### 3.2. Animal Material

The sponge *L. papillosa* was collected from 145 m depth during the 56th scientific cruise of the R/V “Academic Oparin” in June 2019 near Urup Island, Pacific Ocean (45°35′5″ N; 149°47′7″ E) and identified by Mr. B.B. Grebnev (G.B. Elyakov Pacific Institute of Bioorganic Chemistry, Vladivostok, Russia). A voucher specimen is kept under the registration number No. O56-048 in the marine invertebrate collection of the G. B. Elyakov Pacific Institute of Bioorganic Chemistry (Vladivostok, Russia).

### 3.3. Extraction and Isolation

The freshly collected specimens were immediately frozen and stored at −18 °C until use. Animal material (dry weight 54.6 g) was crushed and extracted with EtOH. Concentrated EtOH extract was partitioned between H_2_O and n-BuOH. The n-BuOH-soluble material was concentrated and further fractionated using a reversed-phase flash column chromatography (YMC*Gel ODS-A) eluted with a gradient from H_2_O to EtOH with a step of 20%. The subfraction obtained by elution with 60% EtOH then purified by a multiple reversed-phase HPLC (YMC-Pack ODS-A, 68:32:1 EtOH:H_2_O:1M CH_3_COONH_4_, 1.8 mL/min; Discovery HS-F5-5, 62:38:1 EtOH:H_2_O:1M CH_3_COONH_4_, 2.0 mL/min) to obtain stonikacidin A (**1**) (11.2 mg, 0.02% based on dry weight of the sponge).

### 3.4. Compounds Characterization Data

**Stonikacidin A (1):** colorless amorphous solid, ${\left[\alpha \right]}_{D}^{20}$−4 (c 0.06, MeOH), IR (KBr) ν_max_ 3446, 1703, 1631, 1450, 1416, 1175, 1120, 1074 cm^−1^, Figure S13; UV (MeOH) λ_max_ (log ε) 233 nm (2.37), 270 nm (2.43), Figure S14; ECD (4.99 × 10^−4^, MeOH) λ_max_ (Δε) 265 nm (1.59), 248 nm (−8.20), Figure S15; ^1^H and ^13^C NMR: Table 1, Figures S8–S12; HRESIMS *m*/*z* 800.8683 [M − H]^−^ (calcd for [C_26_H_20_^79^Br_3_N_4_O_11_]^−^ *m*/*z* 800.8684), and *m*/*z* 846.8463 [M_Na–_H + Na]^+^ (calcd for [C_26_H_20_^79^Br_3_N_4_O_11_Na_2_]^+^ *m*/*z* 846.8469), Figures S1–S7.

### 3.5. Alkaline Hydrolysis Followed by Acetylation of Stonikacidin A (***1***)

**Scheme 1:**
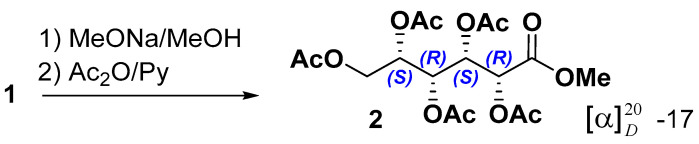
Preparation of methyl-pentaacetyl-L-idonate (**2**) from **1**.

**Scheme 2:**
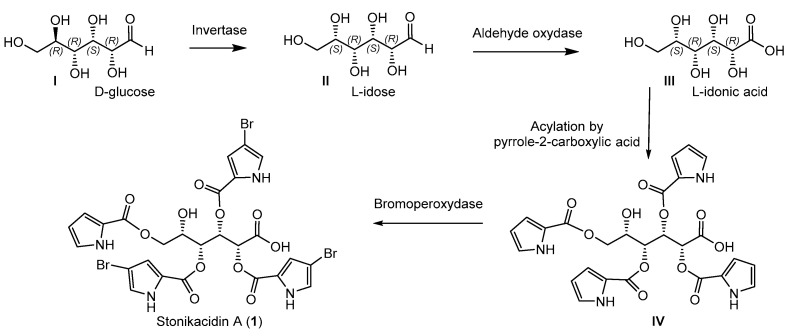
Proposed biosynthetic pathways for stonikacidin A (**1**).

Stonikacidin A (**1**) (4.8 mg) was dissolved in 1500 µL of absolute MeOH, and 600 µL of 0.1 N MeONa was added. After 24 h at room temperature, the reaction mixture was concentrated to dryness under reduced pressure. Then, the dry residue was dissolved in a minimal amount of H_2_O, neutralized with CH_3_COOH, and concentrated. The resulting solid residue was dissolved in 200 μL of pyridine, and 200 μL of Ac_2_O was added. The mixture was left for a day at room temperature, after which it was concentrated in vacuo to dryness. The resulting solid residue was purified using flash column chromatography on a reverse phase YMC*Gel ODS-A sorbent, eluting with a gradient from H_2_O to EtOH with a step of 20%. As a result, the methyl-pentaacetyl-L-idonate (**2**) in a subfraction eluted with 20% EtOH was isolated (Scheme 1, Figures S16 and S18, Table S3).

**Methyl-pentaacetyl-L-idonate (2):**${\left[\alpha \right]}_{D}^{20}$−17 (*c* 0.12, MeOH); ^1^H NMR: Figure S18, Table S3; HRESIMS: [M + Na]^+^ *m/z* 443.1162 (calcd for C_17_H_24_O_12_Na 443.1160), Figure S16.

### 3.6. Synthesis of D-Idose

D-Idose (**3a**) was synthesized by Paulsen acetoxonium rearrangement from 1,2,3,4,6-penta-O-acetyl-β-D-glucopyranose using SbCl_5_ in accordance with known procedure [49] and further deacetylation under Zemplen conditions (sodium methoxide in anhydrous methanol) [50] (Scheme S1).

### 3.7. Synthesis of L-Idose

L-Idose (**3f**) was synthesized from the 1,2-isopropylidene derivative of D-glucuronic acid γ-lactone in accordance with a known procedure [51] (Scheme S2).

### 3.8. Synthesis of Acetylated Methyl Aldonates

The oxidation of monosaccharides (**3a**–**3f**) to potassium salts (**4a**–**4f**) was carried out in accordance with the Moore and Link method [52] (Scheme S3). Acetylation of potassium salts of aldonic acids (**4a**–**4f**) was performed in Ac_2_O with the catalysis of HClO_4_ [53]. Methylation of the carboxylic group for acetylated derivatives (**5a**–**5f**) was carried out in EtOAc with an ethereal solution of diazomethane in accordance with the work of Robbins and Upson [54]. An approximately 0.2 M solution of diazomethane in EtOAc was added dropwise to a solution of acetylated derivatives (**5a**–**5f**) (500 mg, 1.23 mM) in ethylacetate (50 mL) until TLC (Merk silica gel 60, hexane:benzene:acetone (2:1:1 *v*/*v*)) indicated the conversion of starting aldonic acid (R_f_ = 0.30) into a methylated product with R_f_ = 0.65. The reaction mixtures were evaporated in vacuo, and methyl esters (**6b**–**6f**) were purified by crystallization in an Et_2_O-hexane solution. D-Idonic acid derivative (**6a**) was identified as a non-crystallizing syrup and was purified using column chromatography in benzene:ethylacetate (300:5 *v*/*v*). L-Idonic acid derivative (**6f)** was purified using HPLC on Supelco LCSI SEMI-PREP (250 × 10 mm) column in hexane: isopropanol (6:1 *v*/*v*) system.

**Methyl-pentaacetyl-D-idonate (6a):** (slightly yellowish syrup, 476 mg, 92%); ${\left[\alpha \right]}_{D}^{20}$+21 (c 0.12, MeOH); ^1^H and ^13^C NMR: Figures S19 and S20, Table S3; IR (CHCl_3_) ν_max_ 2958, 1755, 1438, 1373, 1245, 1192, 1118, 1051 cm^−1^; HRESIMS: [M + Na]^+^ *m*/*z* calcd for C_17_H_24_O_12_Na 443.1160, found 443.1157.

**Methyl-pentaacetyl-D-gluconate (6b):** (white solid, 460 mg, 89%); ${\left[\alpha \right]}_{D}^{20}$+13 (c 0.22, MeOH); m.p. = 122–123 °C (m.p. 124 °C^4^); ^1^H and ^13^C NMR: Figures S21 and S22, Table S3; IR (CHCl_3_) ν_max_ 2958, 1755, 1438, 1373, 1245, 1192, 1049 cm^−1^; HRESIMS: [M + Na]^+^ *m*/*z* calcd for C_17_H_24_O_12_Na 443.1160, found 443.1159.

**Methyl-pentaacetyl-D-galactonate (6c):** (white solid, 439 mg, 85%); ${\left[\alpha \right]}_{D}^{20}$+14 (c 0.14, MeOH); m.p. = 123–125 °C (m.p. 126–127 °C^4^); ^1^H and ^13^C NMR: Figures S23 and S24, Table S3; IR (CHCl_3_) ν_max_ 2958, 1753, 1438, 1373, 1244, 1192, 1089, 1053 cm^−1^; HRESIMS: [M + Na]^+^ *m*/*z* calcd for C_17_H_24_O_12_Na 443.1160, found 443.1161.

**Methyl-pentaacetyl-D-mannonate (6d):** (white solid, 450 mg, 87%); ${\left[\alpha \right]}_{D}^{20}$+14 (c 0.48, MeOH); m.p. = 79–81 °C; ^1^H and ^13^C NMR: Figures S25 and S26, Table S3; IR (CHCl_3_) ν_max_ 2957, 1753, 1438, 1372, 1244, 1192, 1073, 1043 cm^−1^; HRESIMS: [M + Na]^+^ *m*/*z* calcd for C_17_H_24_O_12_Na 443.1160, found 443.1158.

**Methyl-pentaacetyl-D-talonate (6e):** (white solid, 424 mg, 82%); ${\left[\alpha \right]}_{D}^{20}$+70 (c 0.68, MeOH); m.p. = 66–67 °C (m.p. 78–79 °C^3^); ^1^H and ^13^C NMR: Figures S27 and S28, Table S3; IR (CHCl_3_) ν_max_ 2958, 1754, 1438, 1372, 1242, 1193, 1091, 1048 cm^−1^; HRESIMS: [M + Na]^+^ *m*/*z* calcd for C_17_H_24_O_12_Na 443.1160, found 443.1164.

**Methyl-pentaacetyl-L-idonate (6f):** (slightly yellowish syrup, 466 mg, 92%); ${\left[\alpha \right]}_{D}^{20}$−19 (c 0.11, MeOH); ^1^H and ^13^C NMR: Figures S29 and S30, Table S3; IR (CHCl_3_) ν_max_ 2958, 1755, 1438, 1373, 1245, 1192, 1118, 1051 cm^−1^; HRESIMS: [M + Na]^+^ *m*/*z* calculated for C_17_H_24_O_12_Na 443.1160, found 443.1177, Figure S17.

### 3.9. Determination of Absolute Configurations of the Idonic Acid Residue in Stonikacidin A (***1***)

The absolute configuration of the idonic acid residue in stonikacidin A (**1**) was determined by GC of transesterified **2** with (S)-2-butanol, as described [55]. 0.5 mg of **2** was treated with 100 µL of (S)-2-butanol (Sigma) and concentrated TFA (5 µL), and the sealed vial was heated at 60 °C for 16 h. The solvent was evaporated, and the obtained derivative was acetylated with acetic acid anhydride (100 µL) in pyridine (100 µL) for 20 h at room temperature. After evaporation of solvent, the derivative was compared by GC to authentic samples obtained from methyl-pentaacetyl-D-idonate (**6a**) and methyl-pentaacetyl-L-idonate (**6f**) prepared by the same procedure. The following peaks were detected: (S)-2-butyl-pentaacetyl derivative of stonikacidin A (22.13 min), (S)-2-butyl-pentaacetyl-L-idonate (22.12 min), and (S)-2-butyl-pentaacetyl-D-idonate (22.19 min) (Figure S38).

### 3.10. Bioactivity Assay

#### 3.10.1. Antimicrobial Action

##### Microbial Strains and Antimicrobial Assays

The yeast-like fungi Candida albicans KMM 455 and bacterial strains Staphylococcus aureus ATCC 21,027 and Escherichia coli VKPM (B-7935) (Collection of Marine Microorganisms PIBOC FEB RAS, KMM 644) were cultured on the solid-medium Mueller Hinton broth with agar (16.0 g/L) in a Petri dish at 37 °C for 24 h.

The antimicrobial activity of the compound was tested at concentrations ranging from 100 µM to lower. The effect of the compound on bacterial growth was estimated in accordance with [56]. Gentamicin or nitrofungin was used as a positive control at a concentration of 1 mg/mL, and a 1% DMSO solution in PBS was used as a negative control. The optical density of the bacterial suspension after 18 h was measured at λ = 620 nm.

The effect of the compound on the biofilm formation for 18 h was tested using an MTT reagent (Sigma-Aldrich, St. Louis, MO, USA) in accordance with [57]. The optical density of the obtained solution was measured at λ = 570 nm. MultiskanFS spectrophotometer (Thermo Scientific Inc., Beverly, MA, USA) was used in both assays. The results were calculated as percentages of the control data.

##### Sortase A Activity Inhibition Assay

The enzymatic activity of sortase A from Staphylococcus aureus was determined using a SensoLyte 520 Sortase A Activity Assay Kit Fluorimetric (AnaSpec AS-72229, Ana-Spec, San Jose, CA, USA), according to the manufacturer’s instructions. 4-(Hydroxymercuri)benzoic acid (PCMB) was used as a sortase A enzyme’s activity inhibitor. Fluorescence was measured using a PHERAStar FS plate reader (BMG Labtech, Offenburg, Germany) for 60 min, with a time interval of 5 min at λex = 490 nm and λem = 520 nm. The data were processed using MARS Data Analysis v. 3.01R2 (BMG Labtech, Offenburg, Germany). The results are presented as relative fluorescence units (RFUs).

##### Molecular Docking

The PDB file of sortase A (PDB ID 1T2P) and MurA (PDB ID 1UAE) were obtained from the RCSB Protein Data Bank (https://www.rcsb.org accessed on 1 May 2024) and prepared for docking using the PrepDock package of UCFS Chimera 1.16 software. The chemical structure of 1 was prepared for docking by ChemOffice and checked by the PrepDock package of UCFS Chimera 1.16 software. Docking was conducted on the SwissDock online server (http://www.swissdock.ch accessed on 1 May 2024) using the EADock DSS docking software [58]. The algorithm implies the generation of many binding modes in the vicinity of all target cavities (blind docking) and estimation of their CHARMM energies via the Chemistry at Harvard Macromolecular Mechanics (CHARMM) algorithm [59] for evaluation of the binding modes with the most favorable energies from the Fast Analytical Continuum Treatment of Solvation (FACTS) [60] and, finally, clustering of these binding modes [61].

The predicted building models for the target/ligand pair were visualized and analyzed using UCFS Chimera 1.16 software. Docking parameters such as Gibb’s free energy (ΔG, kcal/mol), energy (kcal/mol), and hydrogen-bonding (H-bond) and hydrophobic interactions were used for the analysis of target/ligand complexes, as described in [62].

#### 3.10.2. Cytotoxic Activity Assay

##### Reagents and Antibodies for Biological Experiments

The following reagents were used for biological experiments: Calcein-AM (BIOZOL, Eching, Germany), Tariquidar (MedChemExpress, Monmouth Junction, NJ, USA), MTT (3-(4,5-dimethylthiazol-2-yl)-2,5-diphenyltetrazolium bromide) (Sigma, Taufkirchen, Germany).

##### Cell Lines and Culture Conditions

The human prostate cancer (22Rv1, LNCaP, VCaP, PC-3, DU145) and non-cancer cell lines (PNT2, RWPE-1, HUVEC, MRC-9, HEK293T) were purchased from ATCC (Manassas, VA, USA) or ECACC (Salisbury, UK). Docetaxel-resistant PC3-DR and DU145-DR were generated by the culturing of PC3 and DU145 cells, respectively, in the sub-lethal concentrations of docetaxel for the time period of 1 year [63]. The culture conditions have been reported previously [64].

##### MTT Assay

Cytotoxic activity was evaluated using MTT assay [65]. In brief, 6000 cells/well were seeded in a 96-well plate in 100 μL/well. The cells were incubated overnight and treated with the investigated compound for 48 h. Then, a solution of MTT reagent (10 mg/mL) was added (10 µL/well), and the plates were incubated for 2 h. The media was aspirated, plates were dried, and DMSO was added to dissolve the formazan crystals. The viability was then measured using calorimetric analysis. The data were analyzed using GraphPad Prism v.9.1.1 (GraphPad Software, San Diego, CA, USA).

##### Statistical Analysis

The generated data were analyzed using GraphPad Prism v.9.1.1 (GraphPad Software) and are represented as mean ± standard deviation (SD). The experiments were performed in biological triplicates (n = 3). A one-way ANOVA test was used for statistical analysis. Statistically significant difference from control is indicated as * (*p* < 0.05).

## 4. Conclusions

Chemical investigation of the marine sponge *L. papillosa* led to the isolation of novel secondary metabolites with antimicrobial activity. The obtained compound, named stonikacidin A (**1**), was proved to be an unprecedented hybrid natural product containing aldonic acid core and bromopyrrole moieties. To date, only a few tannin derivatives, in which polyphenolic residues are attached to gluconic acid, have been reported [66,67,68,69]. To our knowledge, hybrid molecules with an idonic acid residue have not previously been found in either terrestrial or marine natural sources. The structure of stonikacidin A (**1**) is a result of a remarkable confluence of the biogenesis of carbohydrates and bromopyrrole alkaloids. To establish the structure of **1**, a facile method for determining the stereochemistry of aldonic acid core by GC and NMR analysis in obtaining methyl-pentaacetyl-aldonate from **1** in comparison with the same derivatives of standard hexoses was elaborated. The L-form of idonic acid moiety was confirmed by obtaining chiral butyl esters. A biogenetic scheme for the formation of **1** has been proposed. The alkaloid **1** inhibited the growth of *S. aureus* and *E. coli* test strains with IC_50_ 10.9 µM and 22.3 µM, as well as affected the formation of *S. aureus* and *E. coli* biofilms at 14.9 µM and 23.6 µM (IC_50_), respectively. Compound **1** inhibited the activity of sortase A by 38.8–41.4% at 10 µM. It is the first report of antimicrobial compounds from marine sponges of the genus *Lissodendoryx*.

## Figures and Tables

**Table 1:** ^1^H (700 MHz) and ^13^C (175 MHz) NMR Spectroscopic Data for **1** in CD_3_OD.

No.	*δ*_H_, mult (*J* in Hz)	*δ*_C,_ *^a^* Type
1		172.8, C
2	5.65, d (1.6)	73.6, CH
3	6.27, dd (1.6, 9.0)	72.8, CH
4	5.61, dd (1.6, 9.0)	73.0, CH
5	4.27, m	68.4, CH
6a	4.10, dd (6.0, 10.0)	64.6, CH_2_
6b	4.28, m	
2′		123.3, C
3′	6.90, d (1.6)	125.03, *^b^* CH
4′		98.1, C
5′	6.80, d (1.6)	118.9, CH
6′		160.6, C
2″		123.5, C
3″	6.88, d (1.6)	124.7, CH
4″		98.0, C
5″	6.76, d (1.6)	118.8, CH
6″		160.1, C
2‴		123.6, C
3‴	7.01, d (1.6)	125.05, *^b^* CH
4‴		98.3, C
5‴	6.98, d (1.6)	119.0, CH
6‴		160.7, C
2⁗		122.9, C
3⁗	6.67, dd (1.4, 3.6)	117.2, CH
4⁗	6.12, dd (2.4, 3.6)	110.8, CH
5⁗	6.91, dd (1.4, 2.4)	124.9, *^b^* CH
6⁗		162.1, C

*^a^* ^13^C NMR assignment supported by HSQC and HMBC data. ^*b*^ Signals are interchangeable.

**Table 2:** The calculated complexes of **1** with possible enzymatic targets.

Cluster	∆G, kcal/mol	Energy, kcal/mol	H-Bond, Å	Hydrophobic Interactions
PDB ID 1T2P				
0	−8.939546	−24.9548	H18 … Pro163, 2.333	Br … Ala92 Br … Val201, Val166, Val168 Gly167, Ile199, Ala104, Ile182, Leu169, Thr180
1	−8.263234	−9.9639	H4 … Glu105, 2.260	Br … Cys184, Gly192, Trp194 Br … Ala92, Leu97 Ala92, Cys184, Ala104, Ile182, Ile199
PDB ID 1UAE				
28	−7.1534557	6.28514	Lys88 … O, 2.825	Br … Cys115, Br … Cys115, Br … Thr166 Gly118, Lys88, Ala119, Leu111
0	−8.188495	−17.8594	H … Cys115, 2.005 H … Gly114, 2.022	Br … Leu111 Gly114, Lys88, Gly113, Ala119, Gly118

## Data Availability

All relevant data are included in the manuscript or in the Supplementary Data.

## Supplementary Materials

The following supporting information can be downloaded at: https://www.mdpi.com/article/10.3390/md22090396/s1. Description of MS, NMR, and GC data of **1** and biological assays (PDF). Figure S1: (−)HRESIMS spectrum of stonikacidin A (**1**); Figure S2: Isotope pattern of deprotonated molecule ion peak [M − H]− of stonikacidin A (**1**); Table S1: Isotopes of deprotonated molecule ion peak of stonikacidin A (**1**) and corresponding formulas of the major isotopologues; Figure S3: (−)ESIMS/MS spectrum of [M − H]− precursor ion of stonikacidin A (**1**); Figure S4: (+)HRESIMS spectrum of stonikacidin A (**1**); Figure S5: Isotope pattern of [M + 2Na − H]+ ion of stonikacidin A (**1**); Table S2: Isotopes of [M + 2Na − H]+ ion of stonikacidin A (**1**) and corresponding formulas of the major isotopologues; Figure S6: (+)ESIMS/MS spectrum of [M + 2Na − H]+ precursor ion of stonikacidin A (**1**); Figure S7: Fragmentation of [M + 2Na − H]+ precursor ion of stonikacidin A (**1**); Figure S8: ^1^H NMR spectrum of stonikacidin A (**1**) in CD_3_OD (700 MHz); Figure S9: ^13^C NMR spectrum of stonikacidin A (**1**) in CD_3_OD (175 MHz); Figure S10: COSY spectrum of stonikacidin A (**1**) in CD_3_OD (700 MHz); Figure S11: HSQC spectrum of stonikacidin A (**1**) in CD_3_OD (700 MHz); Figure S12: HMBC spectrum of stonikacidin A (**1**) in CD_3_OD (700 MHz); Figure S13: IR spectrum of stonikacidin A (**1**) in KBr; Figure S14: UV spectrum of stonikacidin A (**1**) in MeOH; Figure S15: ECD spectrum of stonikacidin A (**1**) in MeOH; Figure S16: (+)HRESIMS spectrum of methyl-pentaacetyl-L-idonate (**2**); Figure S17: (+)HRESIMS spectrum of methyl-pentaacetyl-L-idonate (**6f**); Figure S18: ^1^H NMR spectrum of methyl-pentaacetyl-L-idonate (**2**) in CDC_l3_ (700 MHz); Figure S20: ^13^C NMR spectrum of methyl-pentaacetyl-D-idonate (**6a**) in CDC_l3_ (175 MHz); Figure S21: ^1^H NMR spectrum of methyl-pentaacetyl-D-gluconate (**6b**) in CDC_l3_ (500 MHz); Figure S22: ^13^C NMR spectrum of methyl-pentaacetyl-D-gluconate (**6b**) in CDC_l3_ (125 MHz); Figure S23: ^1^H NMR spectrum of methyl-pentaacetyl-D-galactonate (**6c**) in CDC_l3_ (500 MHz); Figure S24: ^13^C NMR spectrum of methyl-pentaacetyl-D-galactonate (**6c**) in CDC_l3_ (125 MHz); Figure S25: ^1^H NMR spectrum of methyl-pentaacetyl-D-mannonate (**6d**) in CDC_l3_ (500 MHz); Figure S26: ^13^C NMR spectrum of methyl-pentaacetyl-D-mannonate (**6d**) in CDC_l3_ (125 MHz); Figure S27: ^1^H NMR spectrum of methyl-pentaacetyl-D-talonate (**6e**) in CDC_l3_ (700 MHz); Figure S28: ^13^C NMR spectrum of methyl-pentaacetyl-D-talonate (**6e**) in CDC_l3_ (175 MHz); Figure S29: ^1^H NMR spectrum of methyl-pentaacetyl-L-idonate (**6f**) in CDC_l3_ (500 MHz); Figure S30: ^13^C NMR spectrum of methyl-pentaacetyl-L-idonate (**6f**) in CDC_l3_ (175 MHz); Table S3: NMR data for compounds **2**, and **6a**–**6f**; Figure S31: GC analysis for methyl-pentaacetyl-L-idonate (**2**); Figure S32: GC analysis for methyl-pentaacetyl-D-idonate (**6a**); Figure S33: GC analysis with co-injected of methyl-pentaacetyl-L-idonate (**2**) and methyl-pentaacetyl-D-idonate (**6a**); Figure S34: GC analysis with co-injected of methyl-pentaacetyl-L-idonate (**2**) and methyl-pentaacetyl-D-gluconate (**6b**); Figure S35: GC analysis with co-injected of methyl-pentaacetyl-L-idonate (**2**) and methyl-pentaacetyl-D-galactonate (**6c**); Figure S36: GC analysis with co-injected of methyl-pentaacetyl-L-idonate (**2**) and methyl-pentaacetyl-D-mannonate (**6d**); Figure S37: GC analysis with co-injected of methyl-pentaacetyl-L-idonate (**2**) and methyl-pentaacetyl-D-talonate (**6e**); Figure S38: Fragments of GC chromatograms for: a—pentaacetate of (S)-2-butyl ester of **2**; b—pentaacetate of (S)-2-butyl ester of L-idonate; c—pentaacetate of (S)-2-butyl ester of D-idonate; Figure S39: Effect of stonikacidin A (**1**) on p-glycoprotein activity.
